# How Demographic Factors Impact Attitudes Toward the Recreational Use of Cannabis

**DOI:** 10.7759/cureus.34304

**Published:** 2023-01-28

**Authors:** Thomas A Clobes, Matin Gagnon

**Affiliations:** 1 Health Sciences, California State University Channel Islands, Camarillo, USA; 2 Psychology, California State University Channel Islands, Camarillo, USA

**Keywords:** health policy, stigma, policy, recreational cannabis, medical cannabis, cannabis

## Abstract

While cannabis legalization in the United States has become more commonplace, differences in attitudes toward its use persist. Negative attitudes toward cannabis create barriers to care for those seeking its use for therapeutic purposes. Existing research regarding the attitudes surrounding cannabis is specific to medical cannabis (MC) use or cannabis use in general. To address this gap, the present research sought to explore the demographic factors that influence attitudes toward recreational cannabis, including gender, age, ethnicity, race, level of education, marital status, number of children, the legal status of cannabis in the state of residency, employment status, political party affiliation, political view, and religion. The Recreational Cannabis Attitudes Scale (RCAS) was used to measure participants’ attitudes toward recreational cannabis. A one-way analysis of variance (ANOVA) or one-way Welch ANOVA was used to determine variations in RCAS scores between different demographic groups. Data from 645 participants indicated that gender (*P *= 0.039), employment status (*P *= 0.016), political party affiliation (*P *= 0.002), political view (*P *= 0.0005), the legal status of the state of residence (*P *= 0.003), religion (*P *= 0.0005), and experience with cannabis (*P *= 0.0005) had significant variations between groups regarding attitudes toward recreational cannabis. Understanding the factors that inform attitudes is critical to efforts to destigmatize cannabis use. Education about cannabis is an effective measure in reducing stigma, and paired with demographic information, advocacy efforts can be more accurately targeted.

## Introduction

Cannabis made its appearance in the United States Pharmacopeia in 1850, and late in the nineteenth century, cannabis regularly appeared on pharmacy shelves as an active ingredient in many medications [1]. After the first federal restrictions were placed on cannabis in 1937 with the Marihuana Tax Act, cannabis was removed from the United States Pharmacopeia in 1942. In 1951 and 1956, legal penalties for cannabis possession increased, and by 1970, cannabis was federally outlawed, with the implementation of the Controlled Substances Act. This legislation officially relegated cannabis as a Schedule I substance, classifying it as having no medicinal uses and having a high likelihood of addiction after use [2]. This scheduling of cannabis is still in effect, dramatically limiting research efforts and contributing to a stigma surrounding its use [3,4].

Despite the federal restrictions on cannabis, several states have enacted legislation to improve patient access to the plant. California began this trend in 1996 with the Compassionate Use Act, allowing physicians to recommend cannabis to suffering patients and ensuring that patients had the right to grow or consume cannabis [2]. Since then, several states and districts of the United States have followed suit, with 37 states, the District of Columbia, Guam, Puerto Rico, and the U.S. Virgin Islands having passed legislation that legalizes the use of medical cannabis (MC) [5].

The battle for cannabis legalization, though, is multifaceted. In addition to MC, some states have legalized recreational cannabis (RC) for adult use. As of September 2021, when data collection for this research concluded, 11 states and the District of Columbia have implemented legislation that effectively legalizes RC use for adults in addition to legalized MC [5]. After the November 2022 elections, 10 additional statutes have increased recreational access [6]. Arguments in favor of legalizing RC include economic and sociocultural benefits, as well as the knowledge that it reduces prescription opioid overdoses [7,8]. Conversely, those who oppose the legalization of RC are often concerned about increased motor vehicle accidents due to impaired driving, increased inadvertent access to youth, and increased cannabis use disorder rates [8].

Previous research has shown that race, political party affiliation, political views, religion, state legal status, and cannabis use strongly influence attitudes toward MC [9]. Additionally, previous qualitative research has shown differences in justification for cannabis legalization efforts based on gender [10]. Females described it as less harmful than other substances that are legal, such as alcohol and tobacco, while males reference personal freedom of choice [10]. There is also evidence to suggest that those who have used cannabis at any point in their lives are more likely to have favorable views toward it [9,11]. Much of what is known about the current climate surrounding cannabis use is specific to MC use or cannabis use in general. The present research sought to explore the demographic profiles that influence attitudes toward RC, including gender, age, ethnicity, race, level of education, marital status, number of children, the legal status of cannabis in the state of residency, employment status, political party affiliation, political views, and religion. Given the totality of this previous research, it was predicted these same factors relevant to MC attitudes in addition to gender would be pertinent to contributing toward attitudes regarding RC.

## Materials and methods

Scale selection

This study used the Recreational and Medical Cannabis Scale, which is composed of two separate components, medical and recreational [12]. This research only utilized the Recreational Cannabis Attitudes Scale (RCAS) to measure participants’ attitudes toward RC. RCAS consists of four Likert-scale questions with a possible composite score ranging from 4 to 20. The reliability coefficient of this scale was reported to be 0.91 [12]. Each respondent was also asked a set of demographic and lifestyle questions. In measuring the impact of state legal status on RC attitudes, respondents only provided their state of residence; the researchers coded the state’s legal status manually based on current regulations to avoid potentially inaccurate data being reported by the respondents.

Survey administration

The Qualtrics-moderated survey was made available to United States respondents from February 2021 to September 2021. Respondents were recruited through social media (including both paid advertisements and unpaid posts on the university’s, authors’, and authors’ research associates’ accounts). The primary author’s learning management system was used for his undergraduate courses and distribution to the university’s lifelong learning community. Any survey that was not completed in its entirety was excluded from the analysis.

Data analysis

A one-way analysis of variance (ANOVA) was used to determine variations in RCAS scores between different demographic groups. If the assumption of homogeneity was not met, a one-way Welch ANOVA was run. Post hoc analyses were performed to determine which group(s) within a given independent variable significantly impacted the mean RCAS score differences. For the one-way ANOVA, the post hoc analysis was completed using the Tukey-Kramer post hoc analysis; the Games-Howell post hoc analysis was used for the one-way Welch ANOVA [13,14]. Statistical significance was set at *P *< 0.05.

Ethical approval

The protocols of this study were reviewed and approved by the Institutional Review Board at California State University Channel Islands (#IO5559). Respondents acknowledged informed consent electronically before completing the survey. Due to the sensitive nature of some of the questions, raw data is not being made publicly available.

## Results

After the survey administration period, 673 participants completed some portion of the survey. There were incomplete responses from 28 participants. There were also nine outliers observed from the visual inspection of boxplots, but they were included in the final analysis because they were determined to be valid responses and had a minimal impact on the final means. Therefore, the final analysis included 645 respondents (Table 1).

**Table 1:** Respondents' demographics.

							n	Percentage (%)
Overall mean							645	100
Gender								
					Male		154	23.9
					Female		489	75.8
					Nonbinary		2	0.3
Age (years)								
				18-24			198	30.7
				25-34			113	17.5
				35-44			123	19.1
				45-54			88	13.6
				55-64			74	11.5
				65-74			32	5.0
				75-84			14	2.2
				85 or older			3	0.5
Ethnicity								
			Hispanic				234	36.3
			Non-Hispanic				411	63.7
Race								
			White				392	60.8
			Black or African American				52	8.1
			Asian				42	6.5
			American Indian or Alaska Native				4	0.6
			Native Hawaiian or Pacific Islander				12	1.9
			Other				143	22.2
Marital status								
	Married						266	41.2
	Never married						291	45.1
	Divorced						62	9.6
	Separated						5	0.8
	Widowed						21	3.3
Number of children								
	0						318	49.3
	1						95	14.7
	2						133	20.6
	3 or more						99	15.3
Highest degree								
				Some high school			5	0.8
				High school			234	36.3
				Trade school			59	9.1
				Bachelor's degree			223	34.6
				Master's degree			89	13.8
				Doctoral degree			35	5.4
Employment status								
				Full time			313	48.5
				Part time			98	15.2
				Unemployed looking for work			31	4.8
				Unemployed not looking for work			33	5.1
				Retired			73	11.3
				Student			77	11.9
				Disabled			20	3.1
Political party affiliation								
		Democratic					325	50.4
		Republican					114	17.7
		Independent/No party affiliation					141	21.9
		Libertarian				21		3.3
		Other				14		2.2
		Not registered				30		4.7
Political views								
		Very liberal				122		18.9
		Slightly liberal				161		25.0
		Moderate				257		39.8
		Slightly conservative				77		11.9
		Very conservative				28		4.30
State legal status								
					Illegal	45		7.0
					Medicinal only	71		11.0
					Medicinal and recreational	529		82.0
Religion								
	Catholicism/Christianity					384		59.5
	Judaism					13		2.0
	Islam					7		1.1
	Buddhism					9		1.4
	Hinduism					1		0.2
	Other religion					54		8.4
	No religion					177		27.4
Cannabis use								
	Current or past use					466		72.2
	Never					179		27.8

The mean RCAS score was 13.6 (Table 2). RCAS scores were not normally distributed, as assessed by the Shapiro-Wilk’s test (*P* < 0.05) and visual inspection of the Q-Q plots. The one-way ANOVA was deemed to be appropriate, nonetheless, due to its robustness to deviations from normality [15,16].

**Table 2:** Mean RCAS scores based on demographic variables. RCAS, Recreational Cannabis Attitudes Scale

			n	RCAS	*P*-value
Overall mean			645	13.6	
Gender					
		Male	154	14.3	0.039
		Female	489	13.4	
		Nonbinary	2	14.5	
Age (years)					
		18-24	198	13.3	0.144
		25-34	113	14.5	
		35-44	123	14.0	
		45-54	88	13.3	
		55-64	74	13.7	
		65-74	32	13.0	
		75-84	14	12.0	
		85 or older	3	12.7	
Ethnicity					
		Hispanic	234	13.4	0.318
		Non-Hispanic	411	13.7	
Race					
		White	392	13.6	0.272
		Black or African American	52	14.3	
		Asian	42	12.7	
		American Indian or Alaska Native	4	17.0	
		Native Hawaiian or Pacific Islander	12	14.0	
		Other	143	13.5	
Marital status					
		Married	266	13.2	0.282
		Never married	291	13.8	
		Divorced	62	14.4	
		Separated	5	14.4	
		Widowed	21	13.3	
Number of children					
		0	318	13.8	0.474
		1	95	13.8	
		2	133	13.2	
		3 or more	99	13.3	
Highest degree					
		Some high school	5	14.0	0.988
		High school	234	13.6	
		Trade school	59	13.5	
		Bachelor's degree	223	13.8	
		Master's degree	89	13.4	
		Doctoral degree	35	13.4	
Employment status					
	Full time		313	13.8	0.016
	Part time		98	12.4	
	Unemployed looking for work		31	14.7	
	Unemployed not looking for work		33	14.6	
	Retired		73	13.6	
	Student		77	13.1	
	Disabled		20	14.9	
Political party affiliation					
	Democratic		325	14.0	0.002
	Republican		114	11.8	
	Independent/no party affiliation		141	14.1	
	Libertarian		21	14.0	
	Other		14	14.3	
	Not registered		30	13.9	
Political views					
	Very liberal		122	15.7	0.0005
	Slightly liberal		161	13.4	
	Moderate		257	13.4	
	Slightly conservative		77	11.7	
	Very conservative		28	11.1	
State legal status					
	Illegal		45	14.6	0.003
	Medicinal only		71	14.9	
	Medicinal and recreational		529	13.4	
Religion					
	Catholicism/Christianity		384	12.9	0.0005
	Judaism		13	14.0	
	Islam		7	10.6	
	Buddhism		9	14.3	
	Other religion		54	14.0	
	No religion		177	15.0	
	Hinduism		1	20.0	Excluded from the religion analysis
Cannabis use					
	Current or past use		466	14.6	0.0005
	Never		179	10.9	

Statistically significant variations in RCAS scores were observed between groups based on gender, state legal status, employment status, political party, political views, religion, and current/past cannabis use (Table 2). No significant variations in the RCAS scores were observed between groups based on age, ethnicity, race, education, marital status, and number of children.

Significant results

Gender

**Figure 1:**
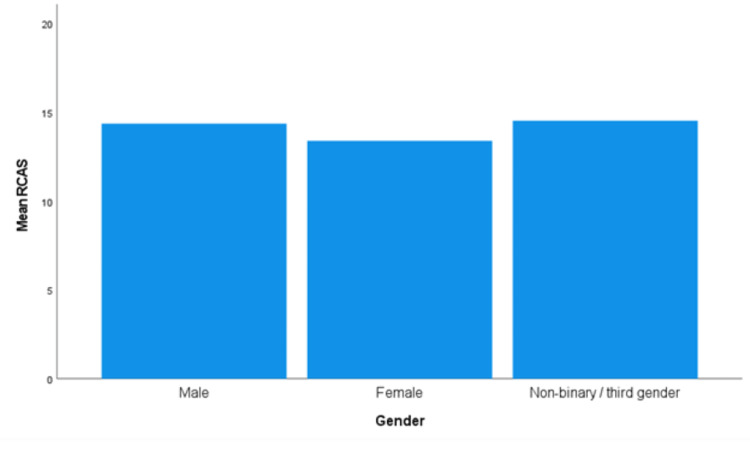
Mean RCAS for gender. RCAS, Recreational Cannabis Attitudes Scale

The assumption of homogeneity of variances was met for gender, as assessed by Levene's test for equality of variances (*P *= 0.497). The RCAS score was significantly higher for men than that for women, *F*(2, 642) = 3.273, and *P *= 0.039 (Figure 1). Men approved of RC (*n *= 154, 14.3 ± 4.10, and 95% confidence interval (CI) 13.7-15.0) more than women (*n *= 489, 13.4 ± 4.1, and 95% CI 13.0-13.7).

State Legal Status

**Figure 2:**
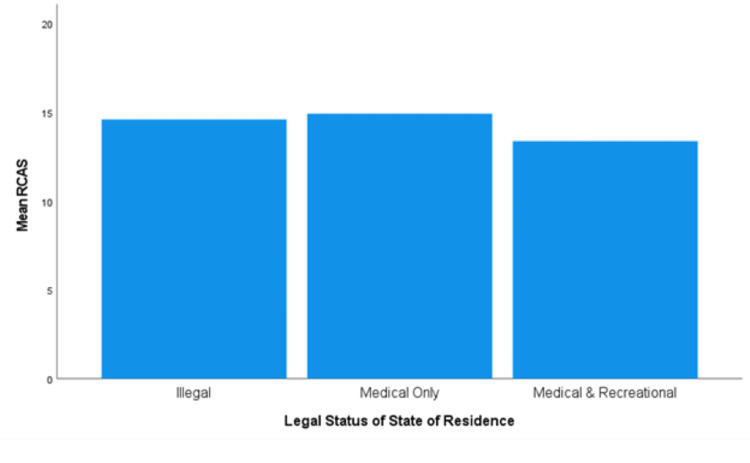
Mean RCAS for legal status of state of residence. RCAS, Recreational Cannabis Attitudes Scale

The assumption of homogeneity of variances was met for state legal status, as assessed by Levene's test for equality of variances (*P *= 0.128). The RCAS score was significantly different based on the legal status of cannabis in the participants’ states of residence, *F*(2, 642) = 5.765, and *P *= 0.003 (Figure 2). The Tukey-Kramer post hoc analysis uncovered a statistically significant lower RCAS for respondents who lived in a state with MC and RC access compared to those with only MC access (*P *= 0.009). Residents of a state with only MC access averaged a significantly higher RCAS (*n *= 71, 14.9 ± 4.03, and 95% CI 14.0-15.9) compared to residents of a state with legal access to MC and RC (*n *= 529, 13.4 ± 4.10, and 95% CI 13.0-13.7). No other state legal status differences were statistically significant.

Employment Status

**Figure 3:**
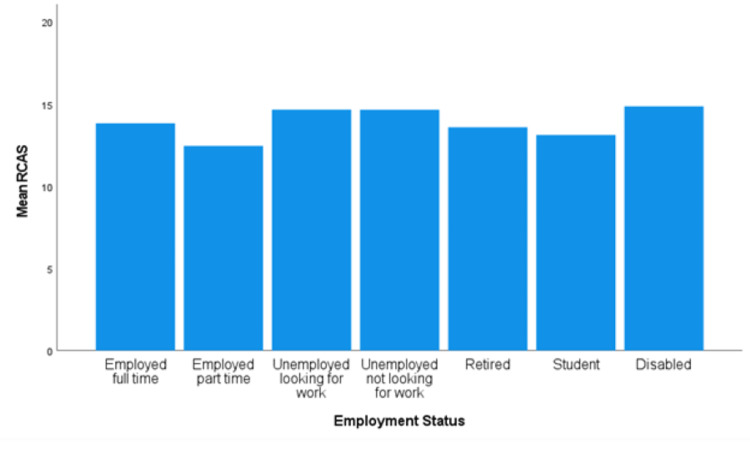
Mean RCAS for employment status. RCAS, Recreational Cannabis Attitudes Scale

The assumption of homogeneity of variances was met for the employment status data, as assessed by Levene's test for equality of variances (*P *= 0.355). The RCAS score varied significantly based on employment status, *F*(6, 638) = 2.622, and *P *= 0.016 (Figure 3). However, the Tukey-Kramer post hoc analysis did not show any significance between the direct employment group comparisons.

Political Party

**Figure 4:**
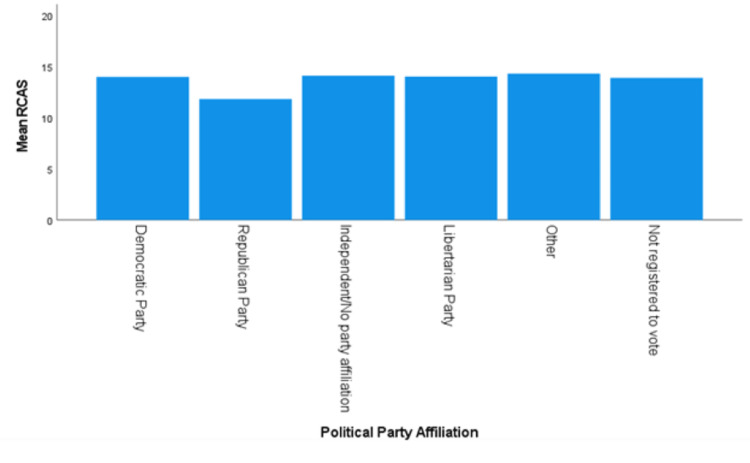
Mean RCAS for political party affiliation. RCAS, Recreational Cannabis Attitudes Scale

The assumption of homogeneity of variances was not met for political party affiliation, as assessed by Levene's test for equality of variances (*P *= 0.042). The RCAS score was significantly different along party affiliation, Welch’s *F*(5, 70.993) = 4.189, and *P *= 0.002 (Figure 4). The Games-Howell post hoc analysis revealed Republicans had statistically lower RCAS than two other groups:

● Democrats’ (*n *= 325) RCAS scores were higher than Republicans’ (*n *= 114) by 2.16 (95% CI 0.750-3.57 and *P *= 0.0005).

● Independent/no party affiliation voters’ (*n *= 141) RCAS scores were higher than Republicans’ (*n *= 114 and *P *= 0.001) by 2.29 (95% CI 0.690-3.89).

No other political affiliation comparisons were significant.

Political Views

**Figure 5:**
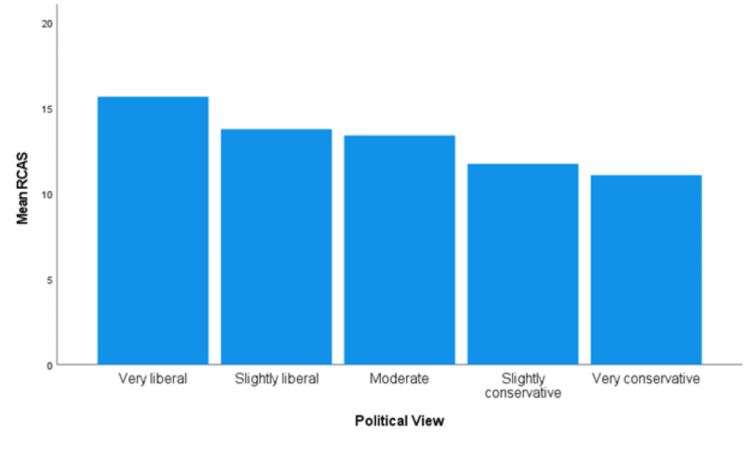
Mean RCAS for political views. RCAS, Recreational Cannabis Attitudes Scale

The assumption of homogeneity of variances was violated for the data on political views, as assessed by Levene's test for equality of variances (*P *< 0.006). There were statistically significant differences in RCAS scores between groups, Welch’s *F*(4, 145.296) = 17.134, and *P *< 0.0005 (Figure 5). The Games-Howell post hoc analysis revealed those who were identified as *very liberal* (*n *= 122) had a statistically significant higher RCAS than every other view:

● 1.89 higher than *slightly liberal* (*n *= 161, 95% CI 0.730-3.04, and *P *< 0.0005)

● 2.26 higher than *moderate* (*n *= 257, 95% CI 1.19-3.33, and *P *< 0.0005)

● 3.92 higher than *slightly conservative* (*n *= 77, 95% CI 2.30-5.54, and *P *< 0.0005)

● 4.58 higher than *very conservative* (*n *= 28, 95% CI 1.98-7.17, and *P *< 0.0005)

Those identifying as slightly liberal (*n *= 161) had a mean RCAS score of 2.03 (95% CI 0.390-3.67, *P* = 0.007) significantly higher than those identifying as slightly conservative (*n *= 77) and 2.69 (95% CI 0.080-5.29) significantly higher than those identifying as very conservative (*n *= 28). Individuals who reported being moderate (*n* = 257) had a mean RCAS score of 1.66 (95% CI 0.0800-3.24 and *P *= 0.034) higher than slightly conservative individuals.

Religion

**Figure 6:**
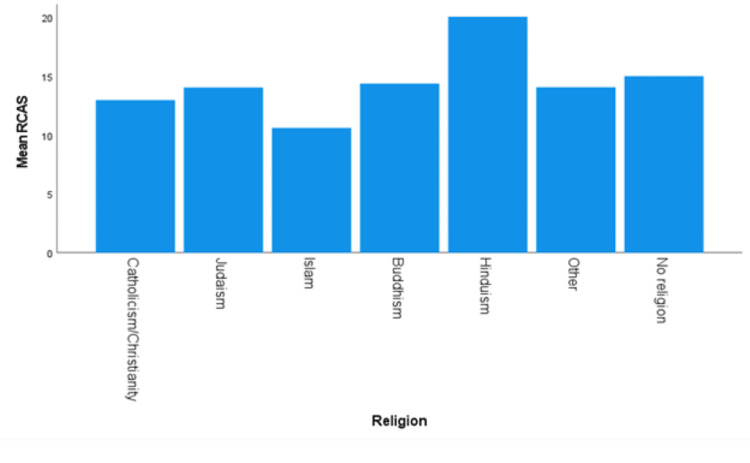
Mean RCAS for religion. RCAS, Recreational Cannabis Attitudes Scale

Levene’s test for equality of variance with the data on religion showed the assumption of homogeneity was met (*P *= 0.353). The differences in mean RCAS scores were significant, *F*(5, 638) = 7.09, and *P *< 0.0005 (Figure 6). The Tukey-Kramer post hoc analysis revealed a higher RCAS in those reporting no religious affiliation (*n *= 177) compared to those reporting being Catholic/Christian (*n *= 384, 2.03, and 95% CI 0.368-3.08), a statistically significant difference (*P *< 0.0005). No other religious group differences were statistically significant. However, the one individual who identified as Hindu was not included in the post hoc analysis because the test of equality means cannot be performed with a group of less than two.

Cannabis Use

**Figure 7:**
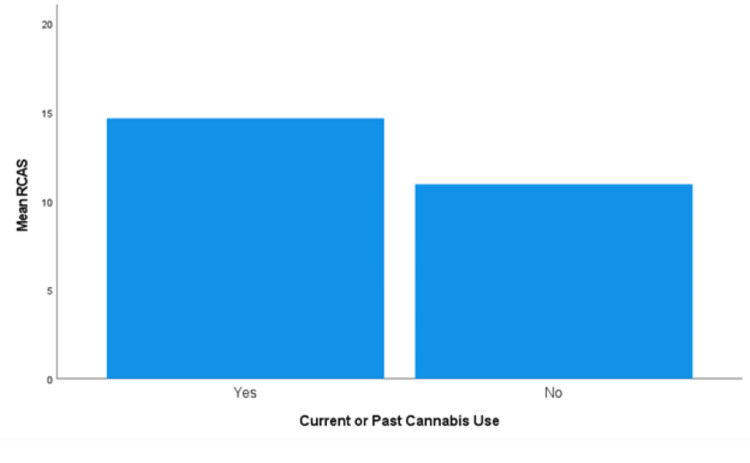
Mean RCAS for current/past users of cannabis versus those who have never used it. RCAS, Recreational Cannabis Attitudes Scale

With the cannabis use data, the assumption of homogeneity of variances was met, as assessed by Levene's test for equality of variances (*P *= 0.765). The mean RCAS was significantly higher for those who have used cannabis (*n *= 466, 14.6, and 95% CI 14.3-15.0) than for those who have never used it (*n *= 179, 10.9, and 95% CI 10.4-11.5), *F*(1, 643) = 122.713, and *P *< 0.0005 (Figure 7).

Nonsignificant results

Age

The assumption of homogeneity of variances was violated, as assessed by Levene's test for equality of variances (*P* = 0.006). There were no statistically significant differences in the RCAS score between different age groups, Welch’s *F*(7, 33.261) = 1.453, and *P *= 0.218.

Ethnicity

Levene’s test for equality of variance showed the assumption of homogeneity was violated (*P *= 0.001). The differences in mean RCAS scores between those identifying as Hispanic and non-Hispanic did not vary significantly, Welch’s *F*(1, 555.325) = 1.097, and *P *= 0.318.

Race

Levene’s test for equality of variance showed the assumption of homogeneity was not met for race (*P *= 0.003). The mean RCAS scores did not vary significantly between self-identified racial groups, Welch’s *F*(5, 25.218) = 0.969, and *P *= 0.456.

Education

Levene's test for equality of variances revealed the data met the assumption of homogeneity (*P *= 0.077). The mean RCAS between different education levels did not vary significantly, *F*(5, 639) = 0.134, and *P *= 0.984.

Marital Status

Regarding marital status, Levene’s test for equality of variance showed the assumption of homogeneity was not met (*P *= 0.003). The mean RCAS scores did not vary significantly regardless of marital status, Welch’s *F*(4, 26.078) = 1.34, and *P *= 0.282.

Number of Children

Levene's test for equality of variances showed the data for the number of children violated the assumption of homogeneity (*P *= 0.048). The mean RCAS score did not vary significantly regardless of the number of children reported by the respondent, Welch’s *F*(3, 233.125) = 0.839, and *P *= 0.474.

## Discussion

Cannabis has had a volatile status in American society for nearly 100 years [1]. Once considered a valuable medical treatment, it was later vilified by government bureaucrats and politicians [2]. Although there has been a resurgence in its medical applications, there are still lingering stigmas surrounding its use [3,4]. This research helped identify demographic factors that shape attitudes toward cannabis.

This current analysis revealed gender, state legal status, employment status, political party, political views, religion, and cannabis use as contributing to attitudes toward RC. A previous analysis found that state legal status, political party, political views, religion, cannabis use, and race were variables shaping attitudes toward MC [9]. While there is considerable overlap between these two lists from the previous and current research, unique factors shaped views separately toward MC and RC (Table 3). Gender and employment status impacted attitudes toward RC but not MC; race contributed to attitudes toward MC but not RC.

**Table 3:** Demographic factors shaping views toward cannabis. Here, × means factors significant for influencing attitudes toward medical cannabis and recreational cannabis.

Demographic factor	Recreational	Medical
Gender	×	
State legal status	×	×
Employment status	×	
Political party	×	×
Political views	×	×
Religion	×	×
Cannabis use	×	×
Race		×

There was a significant difference in attitudes toward RC noted between genders. Men had a more favorable view of the plant than women. This is similar to previous findings, which determined males and females as having different justifications for supporting cannabis legalization [10].

Residents of states with only legal access to MC were more likely to have positive views toward RC than those who live in a state with legal access to MC and RC. This is similar to previous findings of demographic factors that shape attitudes toward MC [9]. This is thought to potentially be a result of the undesirable elements that have been associated with the legalization of RC, most notably complaints about odors, higher referral rates for cannabis use at public schools, increased hospitalizations, and more frequent traffic accidents with the driver being under the influence of cannabis [17-19].

While employment status was determined to have a significant variation in attitudes from the ANOVA, the post hoc analysis did not show any differences. This disagreement between the one-way ANOVA and Tukey-Kramer post hoc can occur because of the distribution of the means [13]. This demographic factor, though, is one determined to contribute to attitudes toward RC but not MC (Table 3) [9]. It has also been noted that cannabis use is associated with the termination of employment, lower income, and problems at work [20].

Political party affiliation was shown to be a significant factor in shaping attitudes toward RC. Republican voters had a significantly lower RCAS score than both Democratic and Independent/no party affiliation voters. Republican voters have been shown to have less favorable views toward MC in prior research, though they also moved toward a more favorable view of MC after being educated on its history, benefits, risks, and medical applications of it [21].

Political views and attitudes toward RC had an inverse relationship. The more conservative one viewed themselves, the less likely they were to have a favorable perception of RC (Figure 5). Similar results were observed with attitudes toward MC [9]. This is also consistent with those who historically supported the war on drugs and efforts to counteract the decriminalization of cannabis [22,23].

Identifying as Christian/Catholic was associated with a less favorable view toward RC than those who held no religious affiliation. There were other religious groups that were associated with less and more favorable views, Islam and Hinduism, respectively, but did not reach statistical significance due to the small number of respondents identifying with those religions. This result of Christians/Catholics having less favorable views of the plant is consistent with other research that found religious college students use cannabis less often, spend less time with cannabis users, and are less likely to support cannabis legalization [24].

Those who had experience with cannabis, medical or recreational, were more likely to have a favorable view of it than those who had never used cannabis. The same findings were found regarding attitudes toward MC [9]. Literature suggests that the favorable attitudes toward cannabis by those who have used it are a result of positive experiences [11]. Those who have personal familiarity with the effects of cannabis are less likely to find it harmful than other substances, such as alcohol.

It is important to understand what influences attitudes toward cannabis for cannabis advocates and patients to work for reform of the current health policy. Given that the legal status of cannabis in a given geography is linked to shifting views of both RC and MC, understanding the specific factors shaping views of RC can help improve attitudes toward MC and the lingering stigma associated with the use of cannabis [3-4,9]. Further, education efforts have been shown to improve attitudes toward MC; although the same is yet to be determined for RC, the data from this and previous research will help cannabis advocates target their education efforts [9,21].

There is a notable limitation of this research. Women, those identifying as white, cannabis users, and those residing in a state with access to both MC and RC were overrepresented in the sample. The high number of full-access state residents and women is due, in part, to the authors residing in California and being affiliated with a university with a high number of female students. This research could be expanded to collect data more uniformly with the population distribution across the country for a more accurate picture of how demographic factors influence attitudes toward RC. Future research would also benefit from larger sample sizes to accurately determine the relationship between Buddhism or Hinduism and cannabis.

## Conclusions

Various factors influence attitudes toward RC. The attitudes toward RC varied between genders, residents of different states, employment status, political party, political view, religion, and experience with cannabis. Understanding what factors contribute to support or opposition of the plant can help cannabis advocates to improve patient access.
